# Supplementary material for: Investigation of the Kinetics and Reaction Mechanism for Photodegradation Tetracycline Antibiotics over Sulfur-Doped Bi2WO6-x/ZnIn2S4 Direct Z-Scheme Heterojunction
Source: Nanomaterials (Basel). 2021 Aug 20;11(8):2123. doi: 10.3390/nano11082123 (PMC8400379; doi:10.3390/nano11082123)
Supplement: Supplementary file 1 [file nanomaterials-11-02123-s001.zip › nanomaterials-1307287-supplementary.pdf]

nanomaterials

MDPI

Supplementary Materils

Investigation of the Kinetics and Reaction Mechanism for Photodegradation Tetracycline Antibiotics over Sulfur-Doped $Bi_2WO_{6-x}/ZnIn_2S_4$ Direct Z-Scheme Heterojunction

Yanbo Jiang [1,2,3], Kai Huang [2,*], Wei Ling [1], Xiandong Wei [1], Yijing Wang [4] and Jun Wang [2,*]

1 Research Center of Wastewater Engineering Treatment & Resource Recovery, Guangxi Beitou Environmental Protection & Water Group Co., Ltd., Nanning 530029, China; yamboo@gxu.edu.cn (Y.J.); lingwei1991@yahoo.com (W.L.); weixiandong@yahoo.com (X.W.)
2 National Engineering Research Center for Non-Food Biorefinery, Guangxi Key Laboratory of Bio-Refinery, Institute of Eco-Environmental Research, Guangxi Academy of Sciences, Nanning 530007, China
3 Institute of Ecological Engineering, Guangxi University, Nanning 530004, China
4 Department of Environment, School of Architectural Engineering, Guangxi University for Nationalities, Nanning 530006, China; jinnywang@gxun.edu.cn
* Correspondence: hwkai@gxas.cn (K.H.); wangjun2016@scau.edu.cn (J.W.); Tel.: +86-771-2503356 (K.H.); +86-771-2503606 (J.W.); Fax: +86-771-2503940 (K.H.)

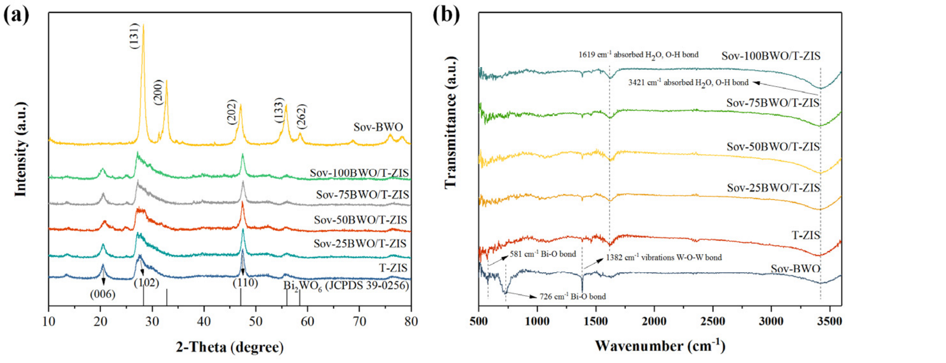

Figure S1. (**a**) XRD patterns and (**b**) FT-IR spectra of the as-fabricated samples (Sov-BWO, T-ZIS, Sov-25BWO/T-ZIS, Sov-50BWO/T-ZIS, Sov-75BWO/T-ZIS, Sov-100BWO/T-ZIS).

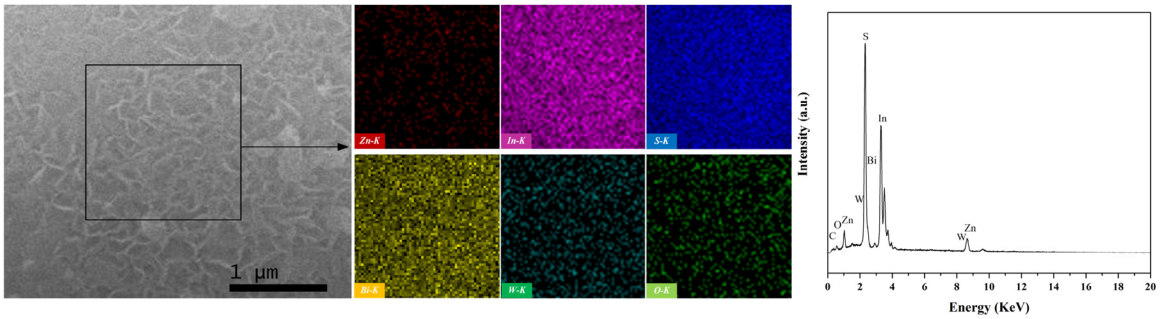

Figure S2. The corresponding EDS spectrum and elemental mapping images of Sov-50BWO/T-ZIS.

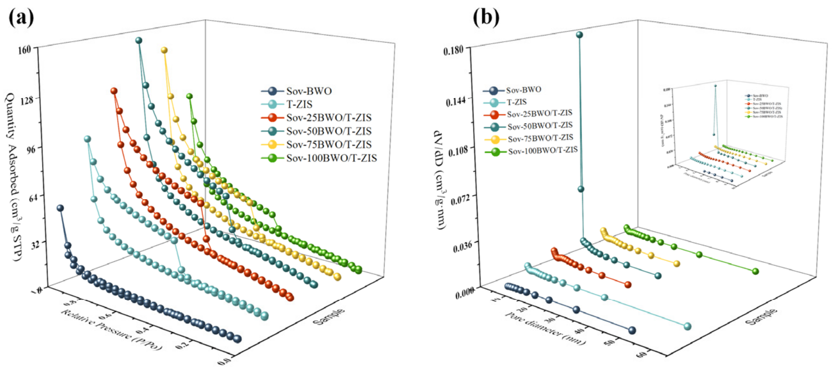

Figure S3. (**a**) N_2 adsorption-desorption isotherms, (**b**) Pore size distributions of prepared samples.

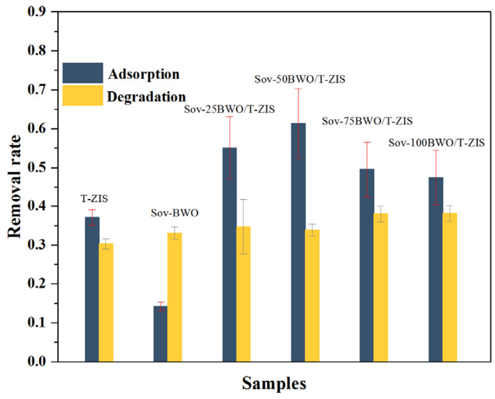

Figure S4. Effect of as-prepared samples on the adsorption and degradation efficiency.

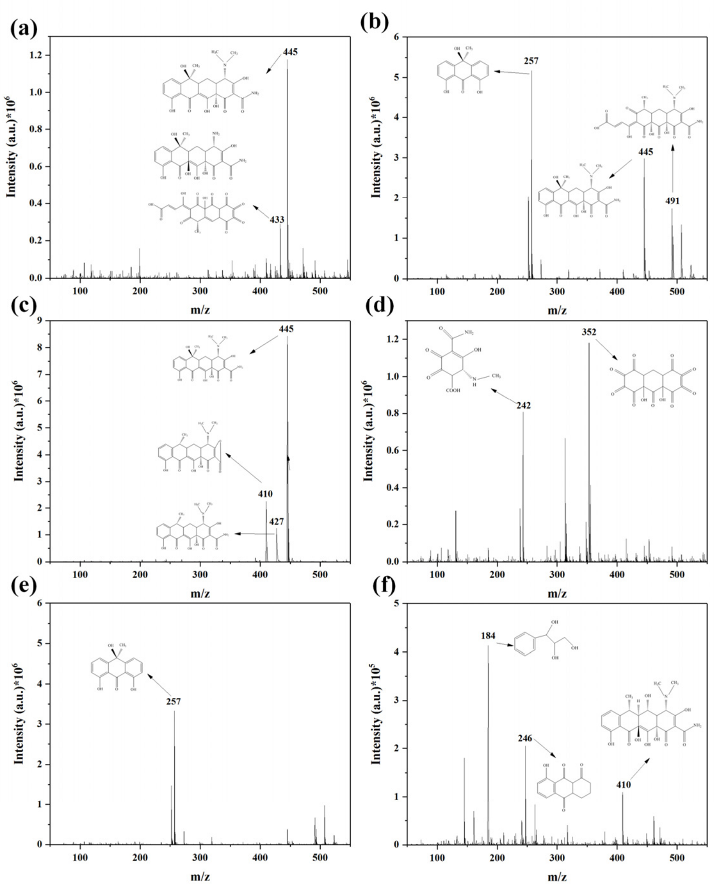

Figure S5. The intermediates analysis of LC–MS for photocatalytic degradation TCH (Represented path: (**a**) PI, PII; (**b**) PIII; (**c**) PII; (**d**–**f**) PI, PII, PIII).

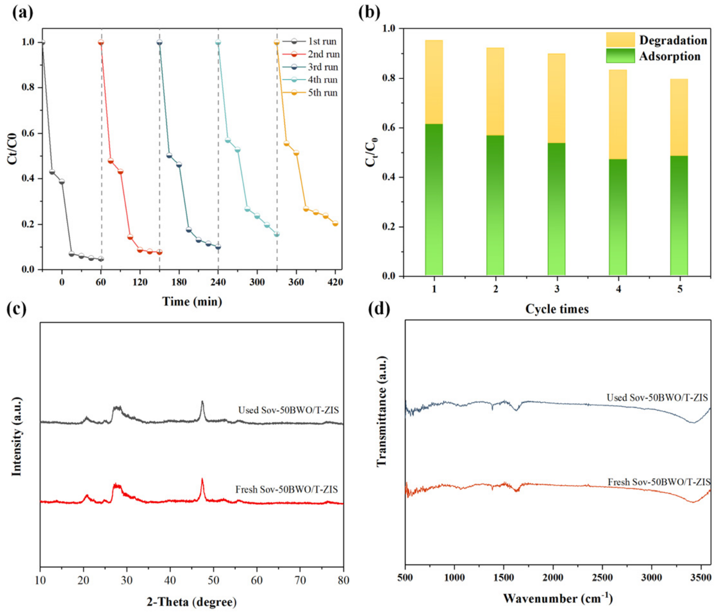

Figure S6. (**a**) Cyclic experiment. (**b**) Comparison of the adsorption and degradation efficiency to test the ability of Sov-50BWO/T-ZIS sample. (**c**) XRD patterns and (**d**) FT-IR spectrum before and after photocatalytic degradation of TCH.

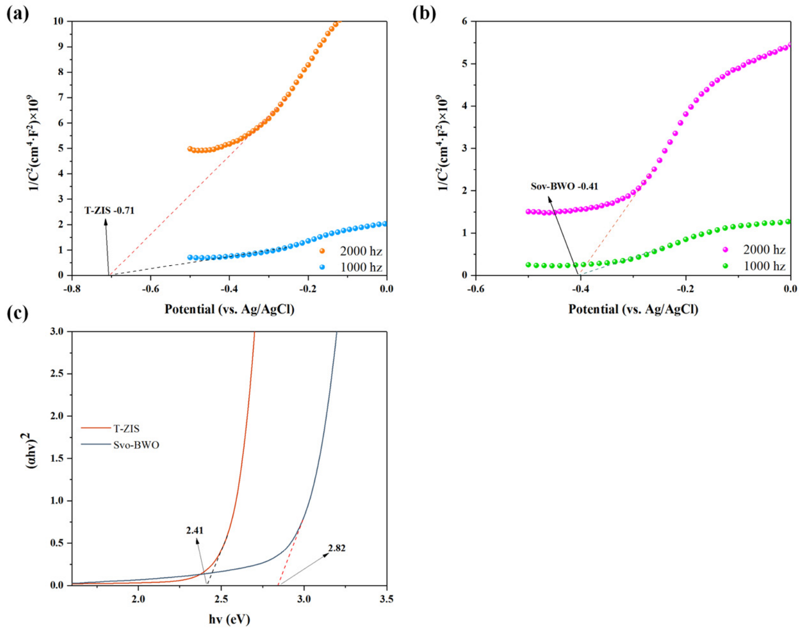

Figure S7. Mott-Schottky plot of (**a**) T-ZIS, (**b**) Sov-BWO in 0.5 M Na_2SO_4 aqueous solution under the frequency of 1000 Hz and 2000 Hz. (**c**) Tauc plots.

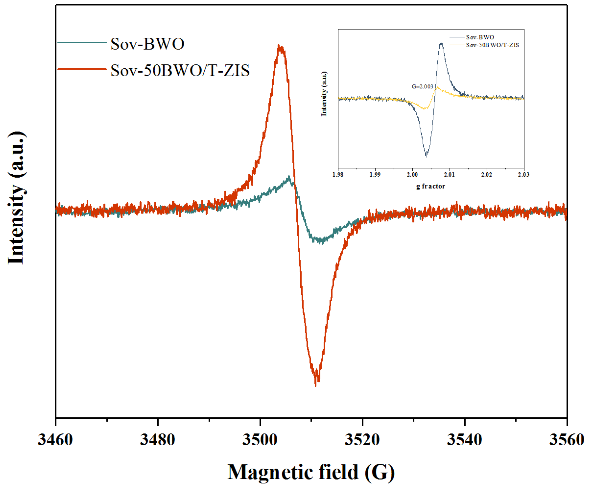

Figure S8. EPR spectra of Sov-BWO and Sov-50BWO/T-ZIS.

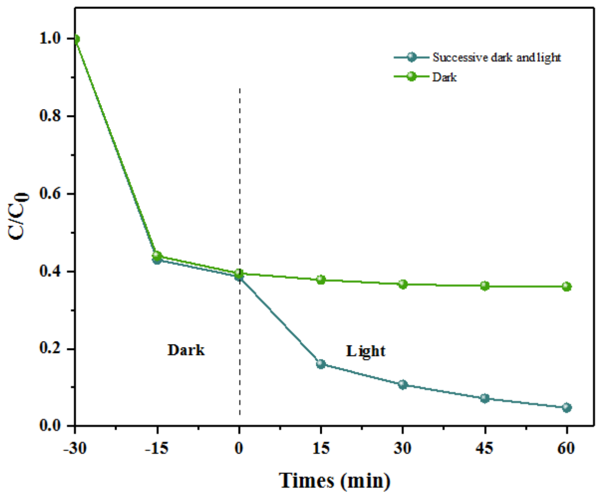

Figure S9. TCH removal curves of Sov-50BWO/T-ZIS under the different irradiation conditions

The result exhibited the superior removal capability by asynchronous adsorption and evidenced the enhanced removal capacity under the visible light irradiation.

Table S1. the as-fabricated samples (Sov-BWO, T-ZIS, Sov-25BWO/T-ZIS, Sov-50BWO/T-ZIS, Sov-75BWO/T-ZIS, Sov-100BWO/T-ZIS) are fitted with R_s, R_p and constant phase elements (CPE).

Sample	R_s (Ω)	R_p (Ω)	CPE (P)
Sov-BWO	9.03	1633.3	1.605
T-ZIS	8.815	1626.1	1.542
Sov-25BWO/T-ZIS	9.024	1370.7	1.589
Sov-50BWO/T-ZIS	8.807	1182.3	1.489
Sov-75BWO/T-ZIS	7.86	1230.8	1.258
Sov-100BWO/T-ZIS	8.567	1441.8	1.525

Table S2. Removal of tetracycline hydrochloride (TCH) over the reported photocatalysys.

Photocatalyst System	Concentration of TCH (mg/L)	Absorption Time (min)	Approximate Absorption Efficiency (%)	Approximate Removal Efficiency in 15 min (%)	Total Removal Efficiency (%)	Ref.
Sov-50BWO/T-ZIS	20	30	61	93	95	This work
15%AgBr/5GO/Bi_2WO_6	20	30	15	88	92	[1]
TiO_2/AB/PS	10	60	20	30	85	[2]
AMM-20	30	30	12	88	90.9	[3]
CFs/g-C_3N_4/BiOBr	20	60	15	40	86.1	[4]
LZO/rGO-3	30	60	32	63	82.1	[5]
AAS/BMO-4	20	30	7	64	92.8	[6]
N-TiO_2/CNONV-2	30	40	19	36	79.9	[7]
BiNPs/g-C_3N_4	10	30	5	77	90.7	[8]
CSM	20	60	13	53	91.6	[9]
CFs/MoS_2/BiOBr	20	60	39	62	92.4	[10]

References

1. Guan, Z.; Li, X.; Wu, Y.; Chen, Z.; Huang, X.; Wang, D.; Yang, Q.; Liu, J.; Tian, S.; Chen, X., et al. AgBr nanoparticles decorated 2D/2D GO/Bi_2WO_6 photocatalyst with enhanced photocatalytic performance for the removal of tetracycline hydrochloride. *Chemical Engineering Journal* **2021**, *410*, 128283, doi:https://doi.org/10.1016/j.cej.2020.128283.
2. Zhang, T.; Liu, Y.; Rao, Y.; Li, X.; Yuan, D.; Tang, S.; Zhao, Q. Enhanced photocatalytic activity of TiO_2 with acetylene black and persulfate for degradation of tetracycline hydrochloride under visible light. *Chemical Engineering Journal* **2020**, *384*, 123350, doi:https://doi.org/10.1016/j.cej.2019.123350.
3. Yang, Z.; Xia, X.; Shao, L.; wang, L.; Liu, Y. Efficient photocatalytic degradation of tetracycline under visible light by Z-scheme Ag_3PO_4/mixed-valence MIL-88A(Fe) heterojunctions: Mechanism insight, degradation pathways and DFT calculation. *Chemical Engineering Journal* **2021**, *410*, 128454, doi:https://doi.org/10.1016/j.cej.2021.128454.
4. Shi, Z.; Zhang, Y.; Shen, X.; Duoerkun, G.; Zhu, B.; Zhang, L.; Li, M.; Chen, Z. Fabrication of g-C_3N_4/BiOBr heterojunctions on carbon fibers as weaveable photocatalyst for degrading tetracycline hydrochloride under visible light. *Chemical Engineering Journal* **2020**, *386*, 124010, doi:https://doi.org/10.1016/j.cej.2020.124010.
5. Wang, Z.; Wang, Y.; Huang, L.; Liu, X.; Han, Y.; Wang, L. $La_2Zr_2O_7$/rGO synthesized by one-step sol-gel method for photocatalytic degradation of tetracycline under visible-light. *Chemical Engineering Journal* **2020**, *384*, 123380, doi:https://doi.org/10.1016/j.cej.2019.123380.
6. Li, S.; Wang, C.; Liu, Y.; Xue, B.; Jiang, W.; Liu, Y.; Mo, L.; Chen, X. Photocatalytic degradation of antibiotics using a novel Ag/Ag_2S/Bi_2MoO_6 plasmonic p-n heterojunction photocatalyst: Mineralization activity, degradation pathways and boosted charge separation mechanism. *Chemical Engineering Journal* **2021**, *415*, 128991, doi:https://doi.org/10.1016/j.cej.2021.128991.
7. Wang, Y.; Rao, L.; Wang, P.; Shi, Z.; Zhang, L. Photocatalytic activity of N-TiO_2/O-doped N vacancy g-C_3N_4 and the intermediates toxicity evaluation under tetracycline hydrochloride and Cr(VI) coexistence environment. *Applied Catalysis B: Environmental* **2020**, *262*, 118308, doi:https://doi.org/10.1016/j.apcatb.2019.118308.
8. Jia, D.; Zhang, Y.; Zhang, X.; Feng, P.; Yang, L.; Ning, R.; Pan, H.; Miao, Y. Facile fabrication of Bi nanoparticle-decorated g-C_3N_4 photocatalysts for effective tetracycline hydrochloride degradation: environmental factors, degradation mechanism, pathways and biotoxicity evaluation. *Environmental Science: Nano* **2021**, *8*, 415-431, doi:10.1039/D0EN01064B.
9. Liang, H.; Lv, C.; Chen, H.; Wu, L.; Hou, X. Facile synthesis of chitosan membranes for visible-light-driven photocatalytic degradation of tetracycline hydrochloride. *RSC Advances* **2020**, *10*, 45171-45179, doi:10.1039/D0RA08358E.
10. Shi, Z.; Zhang, Y.; Duoerkun, G.; Cao, W.; Liu, T.; Zhang, L.; Liu, J.; Li, M.; Chen, Z. Fabrication of MoS_2/BiOBr heterojunctions on carbon fibers as a weaveable photocatalyst for tetracycline hydrochloride degradation and Cr(vi) reduction under visible light. *Environmental Science: Nano* **2020**, *7*, 2708-2722, doi:10.1039/D0EN00551G.
